# Supplementary material for: Tuning electron delocalization of hydrogen-bonded organic framework cathode for high-performance zinc-organic batteries
Source: Nat Commun. 2023 Aug 28;14:5235. doi: 10.1038/s41467-023-40969-5 (PMC10462634; doi:10.1038/s41467-023-40969-5)
Supplement: Supplementary file 1 — Supplementary Information [file 41467_2023_40969_MOESM1_ESM.pdf]

Supplementary Information

Tuning electron delocalization of hydrogen-bonded organic framework cathode for high-performance zinc-organic batteries

Wenda Li[1], Hengyue Xu[2], Hongyi Zhang[1], Facai Wei[1], Lingyan Huang[1], Shanzhe Ke[1], Jianwei Fu[3], Chengbin Jing[1], Jiangong Cheng[4], Shaohua Liu[1]*

[1] State Key Laboratory of Precision Spectroscopy; Engineering Research Center of Nanophotonics & Advanced Instrument, Ministry of Education, School of Physics and Electronic Science, East China Normal University, Shanghai, 200241, P.R. China

[2] Tsinghua Shenzhen International Graduate School, Tsinghua University, Shenzhen 518055, P.R. China

[3] School of Materials Science and Engineering, Zhengzhou University,75 Daxue Road, Zhengzhou, 450052, P. R. China

[4] State Key Lab of Transducer Technology Shanghai Institute of Microsystem and Information Technology, Chinese Academy of Sciences, Shanghai 200050, P. R. China

*Corresponding author: shliu@phy.ecnu.edu.cn (S. L.)

Supplementary calculations

Activation Energy.

Activation energy calculation. The ln (R_{ct}^{-1}) values were plotted vs. 1000/T and linear fit was carried out according to the Arrhenius equation of $\ln(Rct^{-1}) = -E_a/RT + C$, where C is constant under a stable experimental condition, R is the gas constant and T is temperature. The E_a represents the activation energy for charge transfer and was calculated from the slope of the fitted line. Similarly, the activation energy Ea for diffusion was calculated from the Arrhenius equation with diffusion coefficient (D) of $\ln(D) = -E_a/RT + C'$. D was calculated from GITT based on the following equation:

$$D = \frac{4L^2}{\pi\tau}\left(\frac{\Delta E_s}{\Delta E_t}\right)^2 \quad (1)$$

Where τ is the relaxation time, ΔE s is the steady-state potential change after a single pulse, and ΔEt is the potential change during a pulse after eliminating iR drop. The diffusion length L was measured by the geometric thickness of the cathode. Since L was a constant, the value would not affect the activation energy obtained from the slope of the Arrhenius equation. The linearity between cell voltage and $t_{1/2}$ during titration was checked to confirm the applicability of the equation.

Proton Capacity Contribution.

The capacity contribution from H^+ and Zn^{2+} was calculated based on the inductively coupled plasma atomic emission spectroscopy and element analysis. The contents of Zn and S elements in fully discharged BBQPH cathodes are 82.14 wt% and 4.53 wt%, respectively.

The molar ratio of coordinated Zn^{2+} and $Zn_4SO_4(OH)_6{\cdot}xH_2O$ is

$$\left(\frac{82.14}{65.39} - \frac{4.53}{32} \times 4\right) : \left(\frac{4.53}{32}\right) = 0.69 : 0.14 \quad (2)$$

The molar ratio of coordinated Zn^{2+} and H^+ is

$$\left(\frac{82.14}{65.39} - \frac{4.53}{32} \times 4\right) : \left(\frac{4.53}{32} \times 6\right) = 0.69 : 0.85 \quad (3)$$

The capacity contribution ratio of coordinated Zn^{2+} and H^+ is

$$\left(\frac{82.14}{65.39} - \frac{4.53}{32} \times 4\right) : \left(\frac{4.53}{32} \times 6\right) = 0.69 : 0.85 \quad (4)$$

Therefore, the capacity contribution of H^+ and Zn^{2+} in HBOSs is 55% and 45%, respectively.

Redox Electron Transfer Number.

The theoretical capacities (C_m, mAh g^{-1}) of BBQPH and BBQPD were calculated according to the

following equation:

$$C_m = \frac{n \times F}{3.6\ M} \qquad (5)$$

The electron transfer number (n) during the redox reaction process was calculated based on the following form:

$$n = \frac{3.6 C_m \times M}{F} \qquad (6)$$

where M is the molar mass of organic molecules (g mol^{-1}), and F is a constant (96485 C mol^{-1}).

Based on the GCD profile in Figure 4f, the discharge capacities at the three discharging voltage plateaus are 196 (step 1), 121 (step 2) and 178 mAh g^{-1} (step 3), respectively. Correspondingly, the electron-transfer numbers are calculated to be 4 and 2.5, and 3.5, which corresponds to the uptake of $2Zn^{2+}$ and $6H^+$.

Calculation of potential difference based on Nernst Equation

The electrochemical reaction of BBQPH electrode in aqueous H_2SO_4 could be expressed as follows:

$$\text{BBQPH} + n\text{H}^+ + n\text{e}^- \Rightarrow \text{BBQPH-H}_n$$

Nernst Equation:

$$\varphi = \varphi 0 + \frac{2.303RT}{nF} lg \frac{[\text{BBQPH}][\text{H}^+]^n}{[\text{BBQPH}-\text{Hn}]} = \varphi 0 + \frac{0.0529}{n} lg \frac{[\text{BBQPH}][\text{H}^+]^n}{[\text{BBQPH}-\text{Hn}]} \qquad (7)$$

Where φ is electrode potential; φ^0 is the standard potential; R is the ideal gas constant: 8.314 J K^{-1} mol^{-1}; T is the temperature: 298.15 K; n is the electron transfer numbers; F is the Faraday constant: 96500 C mol^{-1}. The activity of solid BBQPH and $BBQPH\text{-}H_n$ is considered as 1, thus the equation can be further simplified as:

$$\varphi = \varphi 0 + \frac{0.0529}{n} \text{lg}[\text{H}^+]^n = \varphi 0 + 0.0529\text{lg}[\text{H}^+]^n \qquad (8)$$

Therefore, the potential difference of BBQPH in a 0.05 M H_2SO_4 solution and in a dilute H_2SO_4 electrolyte with a pH value of about 3.7 (the same pH value in 3M $ZnSO_4$ aqueous solution) can be calculated as:

$$\Delta\varphi = 0.0529\text{lg}\frac{[\text{H}^+]1}{[\text{H}^+]2} = 0.0529\text{lg}\frac{0.1}{10^{-3.7}} = 0.14\ \text{V}$$

Capacitive Contribution.

The ion transport kinetics of Zn//BBQPH batteries were investigated based on the relationship:

$$i = k \times v^{b} \qquad (9)$$

where k and b are constants, i is the current density, and v refers to the scan rate. When the power exponent b value is close to 0.5, it indicates a diffusion-controlled process, while a b-value of 1.0 identifies a surface-controlled reaction.

Dunn's method was applied to analyze the capacitance contribution from the capacitive process (rapid surface redox reactions) and the diffusion-controlled process.

Quantitative analysis for capacitive contribution can be through the following equation:

$$i = k_1 v + k_2 v^{1/2} \qquad (10)$$

where k_1 and k_2 are constants, $k_1 v$ and $k_2 v^{1/2}$ represent the current density contributed by the fast-capacitive process and diffusion-controlled process, respectively. Dividing $v^{1/2}$ into both sides of the above equation yields:

$$i/v^{1/2} = k_1 v^{1/2} + k_2 \qquad (11)$$

Therefore, $i/v^{1/2}$ and $v^{1/2}$ are expected to give a linear relationship, two capacity contributions can be distinguished by linear fitting. The slope corresponds to k_1 and the y-intercept equals k_2. By repeating the above steps for other potentials and scan rates, the respective capacity contribution from the surface-dominated process and a diffusion-limited process can be quantitatively calculated.

Supplementary figures:

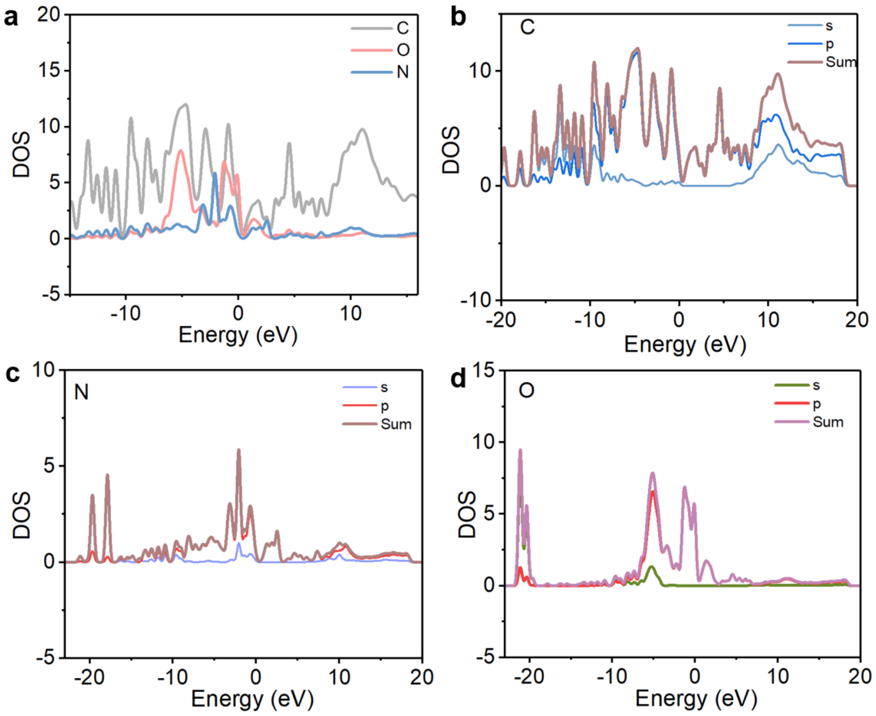

Supplementary Figure 1. The electronic property investigation of BBQPH. (a) The partial density of states (pDOSs) of BBQPH. The detailed pDOSs are based on the different orbits of (b) C (c) N and (d) O elements in BBQPH.

<u>Note</u>: In the pDOS Spectra of BBQPH, the O element plays a crucial role in the conductivity aspects of molecules (The Fermi level is not zero).

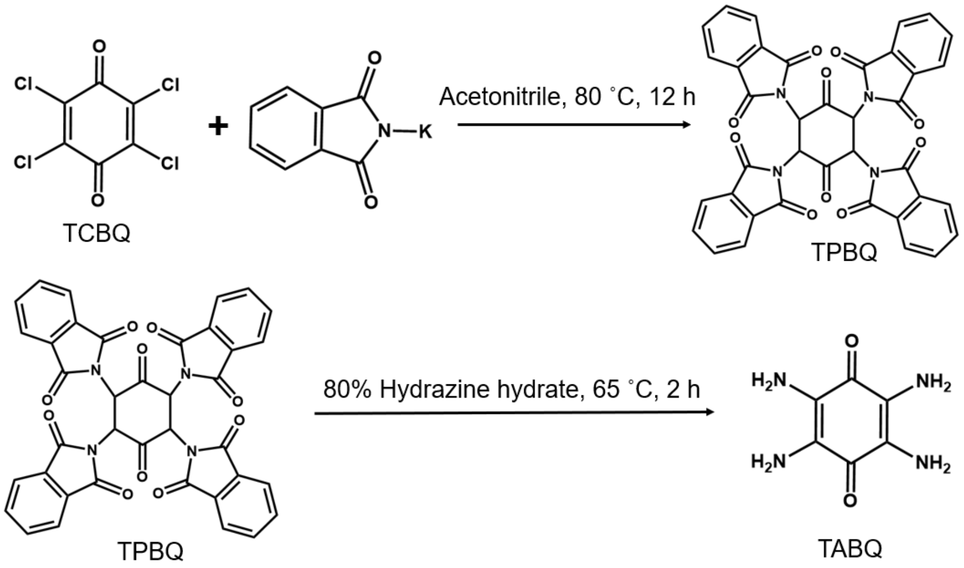

Supplementary Figure 2. Synthetic route of tetraamino-p-benzoquinone (TABQ).

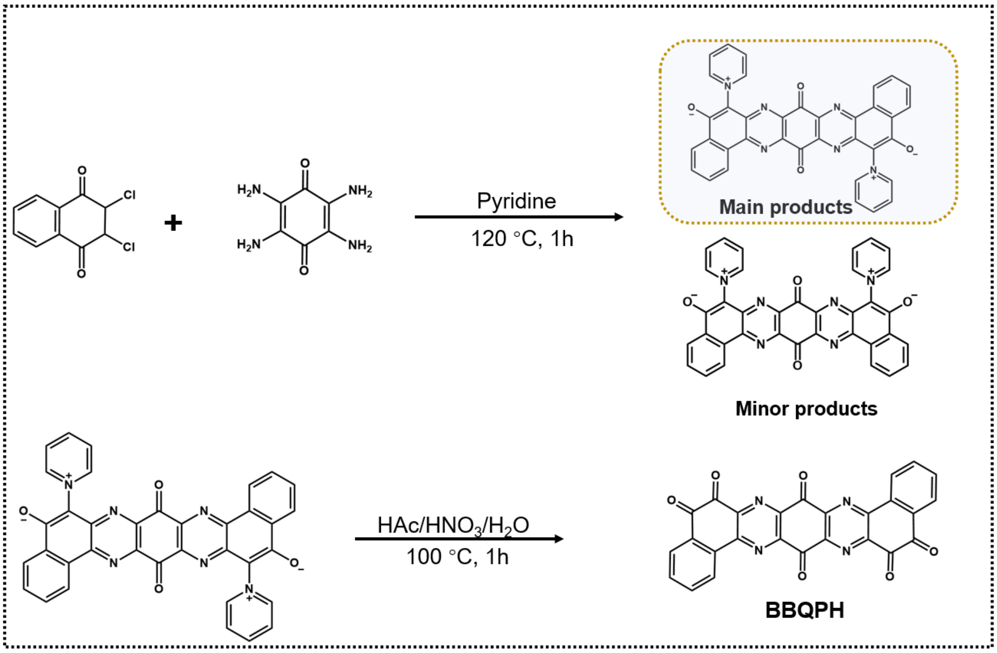

Supplementary Figure 3. **Synthetic route of the BBQPH.** The BBQPH was prepared based on a simple dehydration condensation reaction together with an oxidation reaction.

Note: The obtained molecular configuration is inclined to the centrosymmetric structure due to the steric hindrance and para-energy minimum effect.

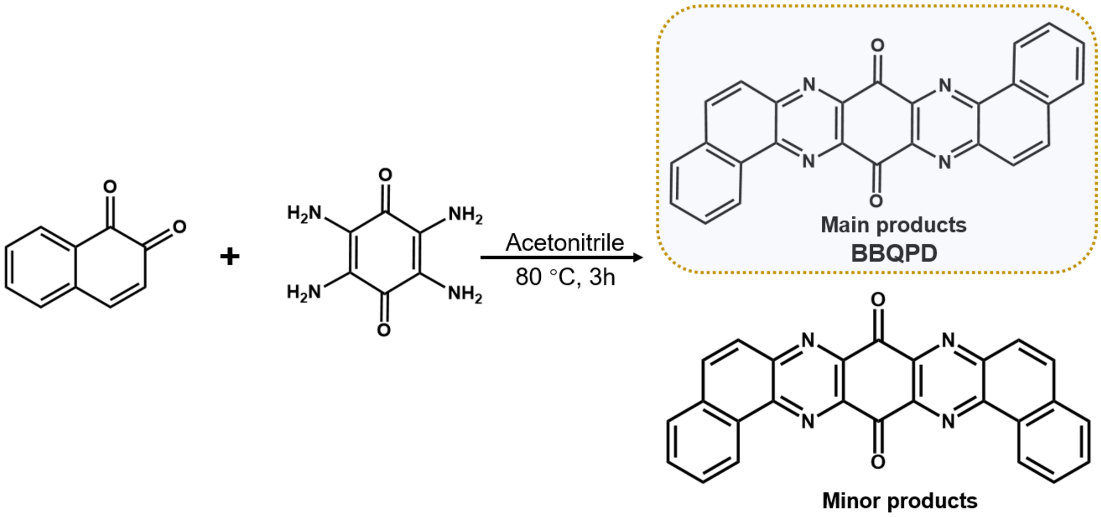

Supplementary Figure 4. **Synthetic route of the BBQPD.**

Note: The obtained molecular configuration is inclined to the centrosymmetric structure due to the steric hindrance and para-energy minimum effect.

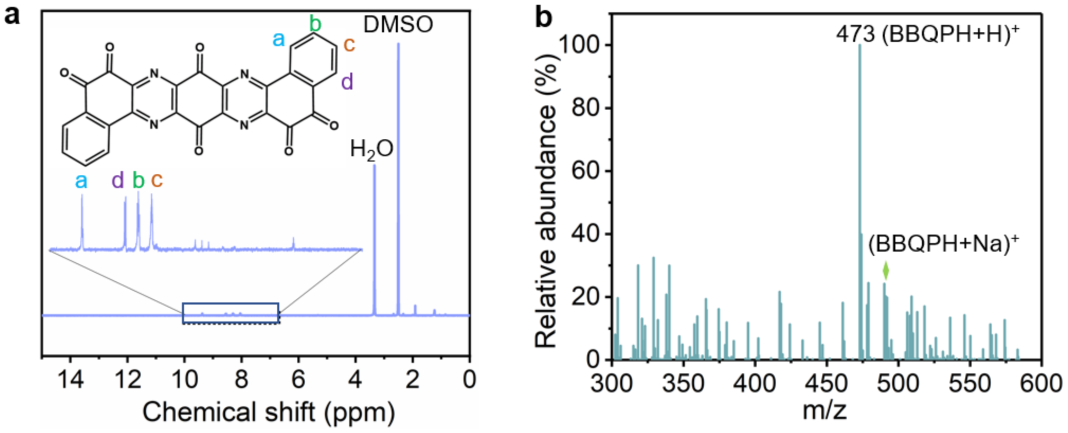

Supplementary Figure 5. Molecular structure characterizations. (a) The ^{1}H NMR spectroscopy of prepared BBQPH. (b) The high-resolution mass spectrometry of prepared BBQPH.

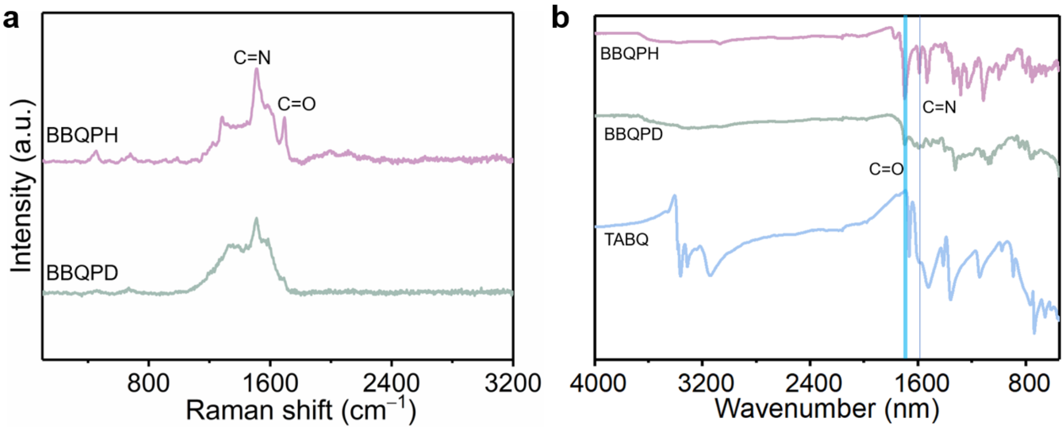

Supplementary Figure 6. Component analysis of the BBQPH and BBQPD. The FTIR (a) and Raman spectrums (b) of BBQPH and BBQPD, respectively.

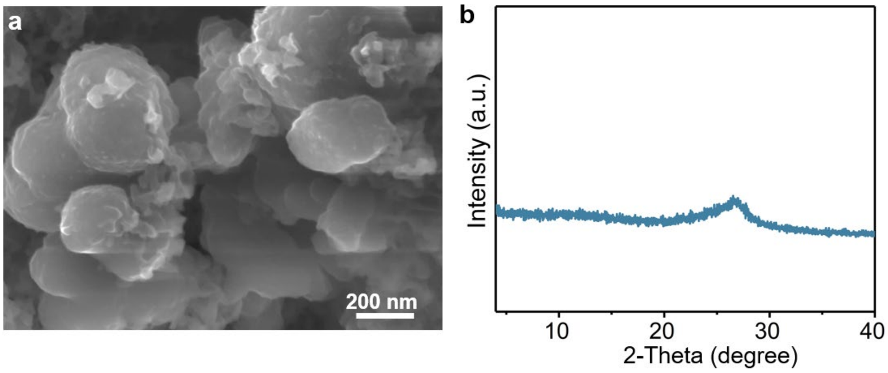

Supplementary Figure 7. Morphology and structure characterizations of BBQPD. (a) The SEM image of the BBQPD. (b) The XRD pattern of the BBQPD.

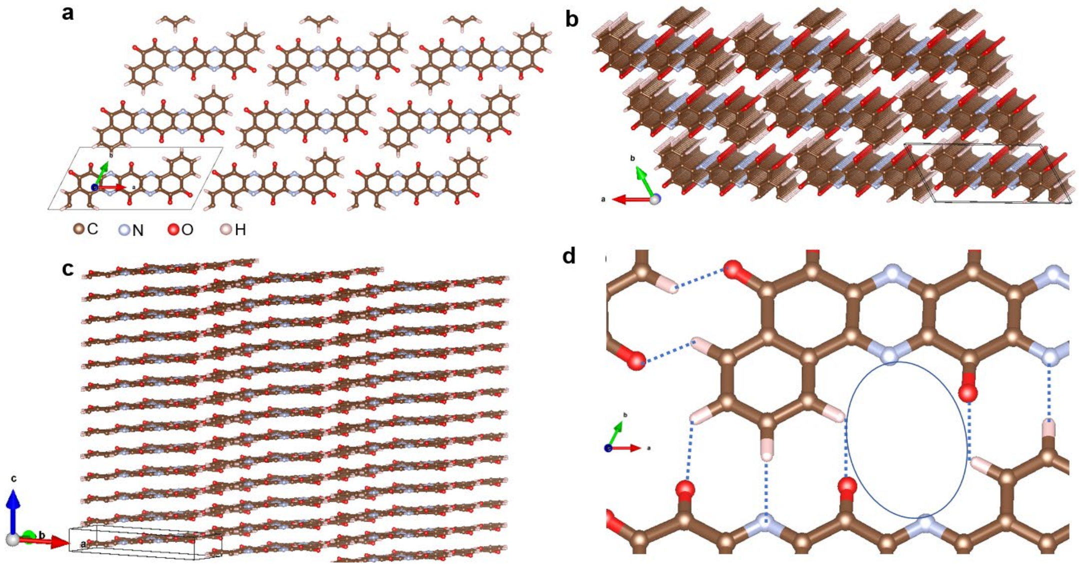

Supplementary Figure 8. Simulated stacking structure of BBQPH hydrogen-bonded organic framework. (a-d) The calculated crystal structure of BBQPH was observed through different angles.

Note: The full-conjugated structure and the rich C=O and C=N groups of BBQPH molecule make it connected to four adjacent BBQPH molecules by multiple hydrogen bonding between weak hydrogen donor C-H groups and strong hydrogen bond acceptor C=O/C=N groups (C=O···H/ C=N···H), forming 2D planar supramolecular structure. The bond lengths of the hydrogen bond between the hydrogen atom in C-H and the oxygen atom in the carbonyl group were about 2.35 Å and 2.40 Å, which are consistent with the typical hydrogen bonds in this work.

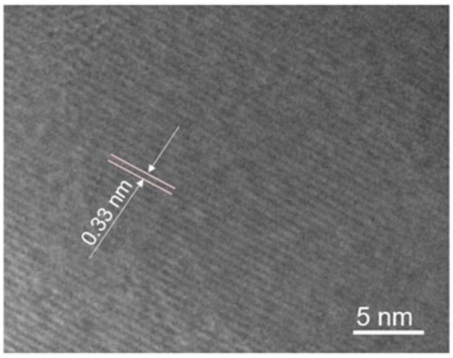

Supplementary Figure 9. High-resolution transmission electron microscopy (HRTEM) image of the BBQPH.

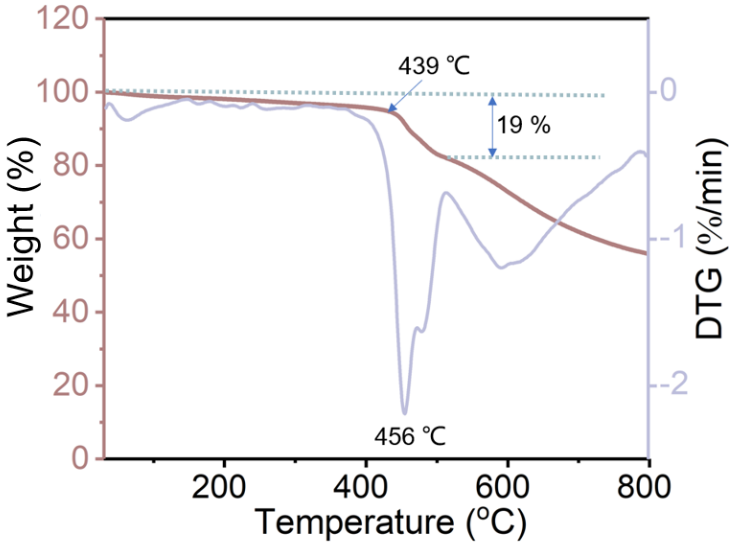

Supplementary Figure 10. The thermogravimetry analysis (TGA) curve of the BBQPH material.

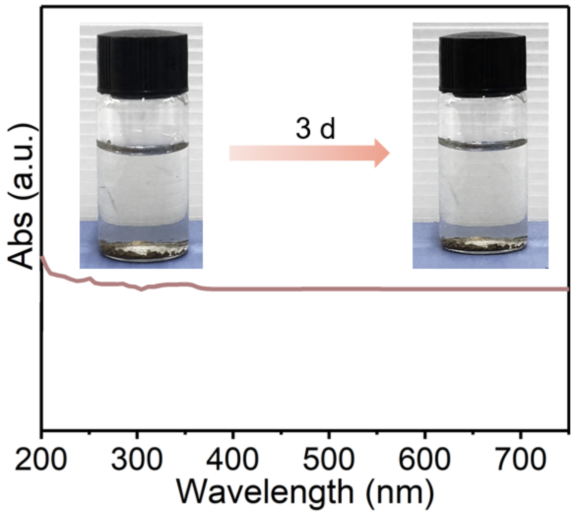

Supplementary Figure 11. The photograph of the BBQPH immersed in water for 3 days and the corresponding UV-Vis spectroscopy.

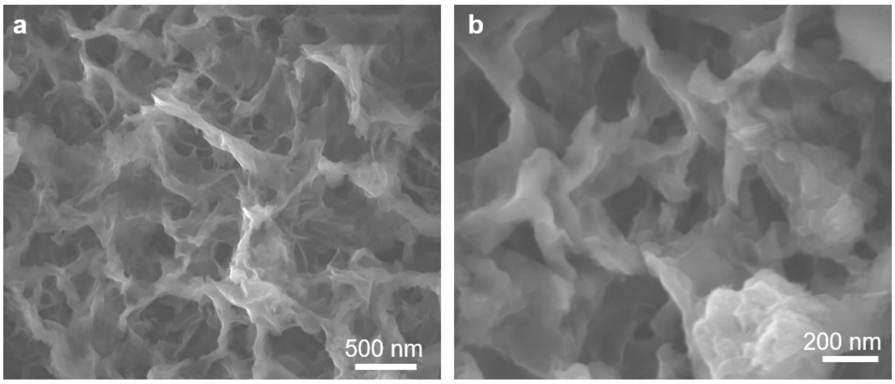

Supplementary Figure 12. Morphology characterization of the BBQPH. (a-b) SEM images of freshly prepared BBQPH.

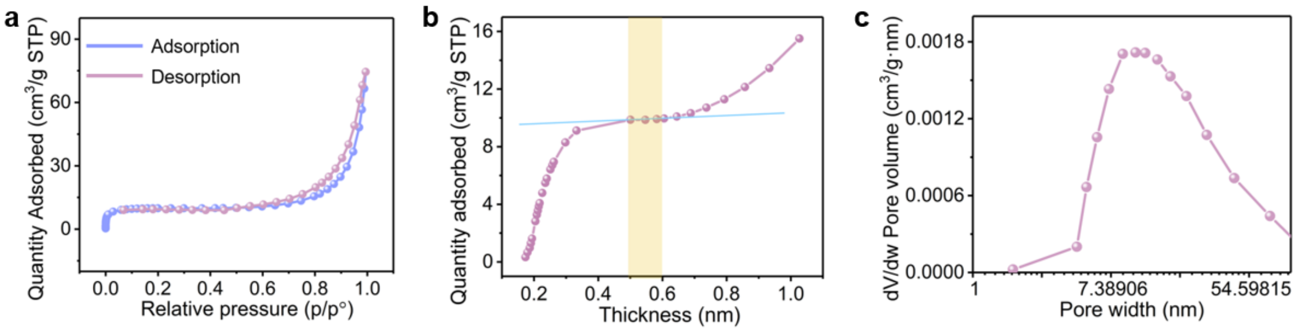

Supplementary Figure 13. Specific surface area and pore size analysis. (a) N_2 adsorption/desorption isotherms of the BBQPH. (b) The micropores size distribution curves by t-plot analyses. (c) The mesopores size distribution curves by Barret-Joyner-Halenda (BJH) analyses.

Note: The BBQPH demonstrates a specific surface area of 68.12 m^2 g^{-1} combined with a multistage pore size of 0.54 nm and 18 nm. The t-Plot results show that the micropores (0.54 nm) exist inside the BBQPH hydrogen-bonded organic framework. While the mesoporous (18 nm) arises from the accumulation of sponge structures.

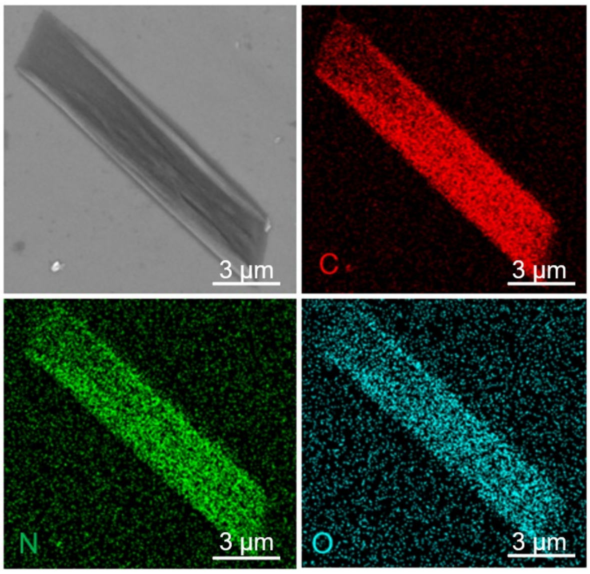

Supplementary Figure 14. SEM image of the modulated BBQPH crystal and corresponding EDS element distribution maps.

Note: The BBQPH single-crystal was modulated by trifluoroacetic acid and chloroform with proper ratio. In detail, 50 mg BBQPH was dissolved in a mixed solvent of chloroform/ trifluoroacetic acid (4: 1 in vol./vol.) and allowed the solvent to evaporate slowly.

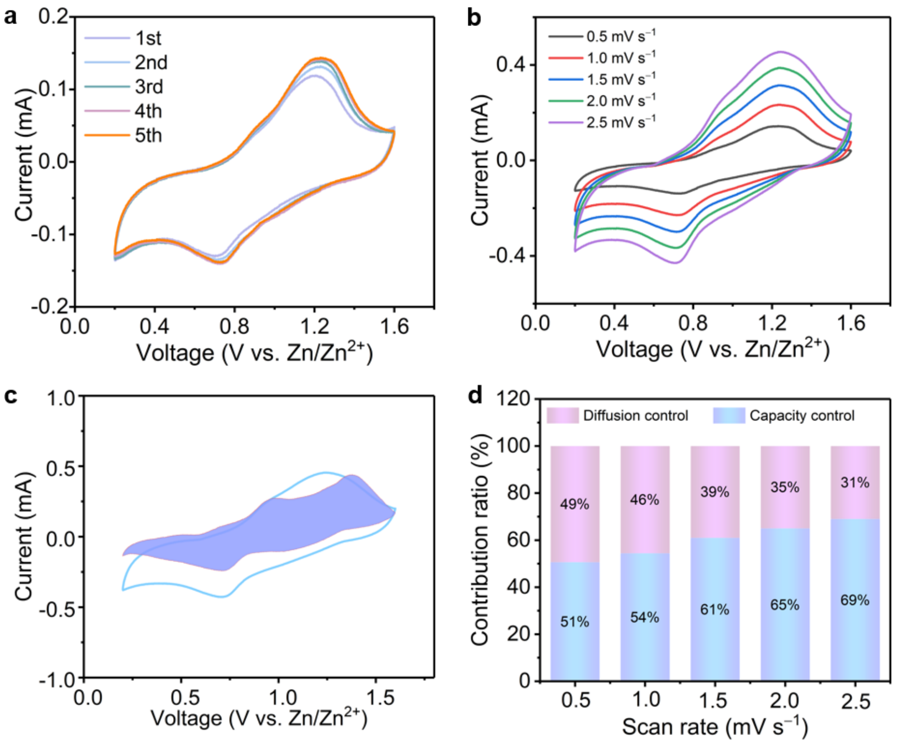

Supplementary Figure 15. Capacitance contributions calculation of BBQPD. (a) CV curves of BBQPD electrode at 0.5 mV s^{-1}. (b) CV curves at various scan rates. (c) Capacitive contribution at 0.5 mV s^{-1}. (d) Ratios of capacitive and diffusion-controlled contribution at various scan rates.

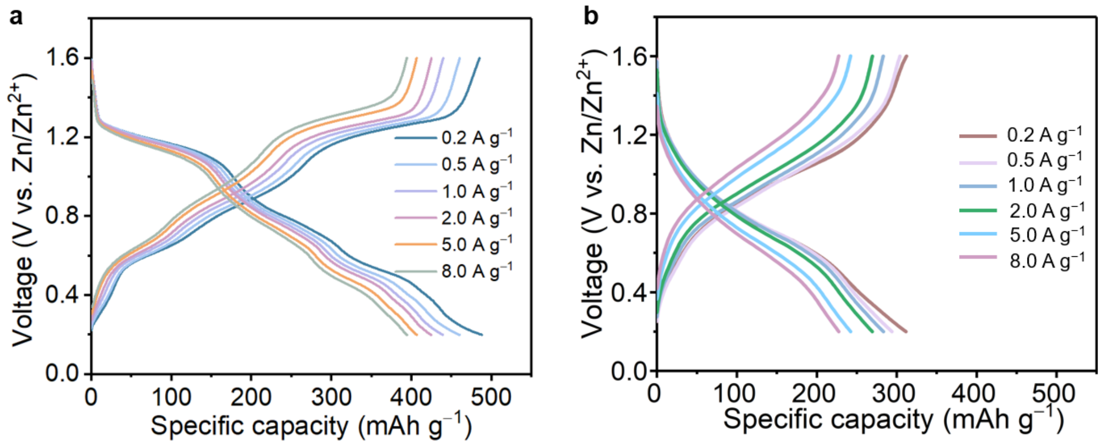

Supplementary Figure 16. Comparison of rate performances of ZOBs. Voltage-capacity profiles of (a) BBQPH and (b) BBQPD electrodes.

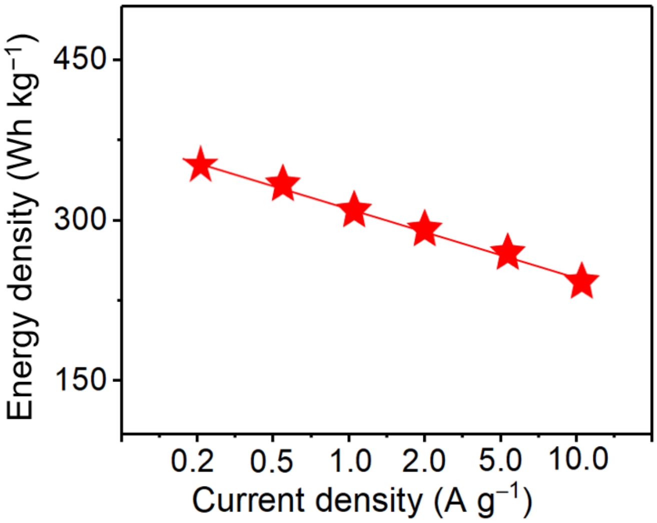

Supplementary Figure 17. Ragone plots of the Zn//BBQPH battery based on the mass loading of BBQPH in the cathode.

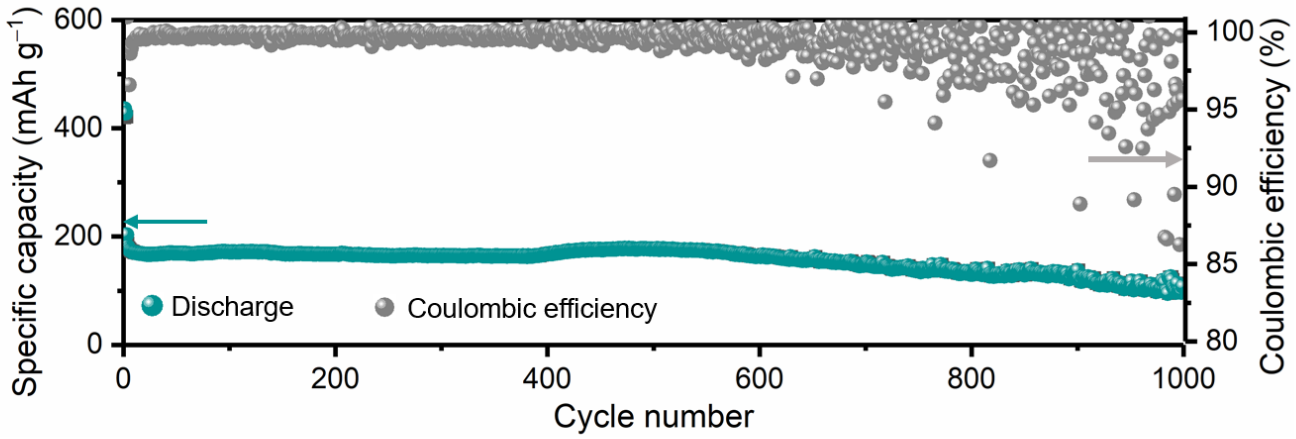

Supplementary Figure 18. The long cycling performance of BBQPD for ZOBs at 5.0 A g^{-1}.

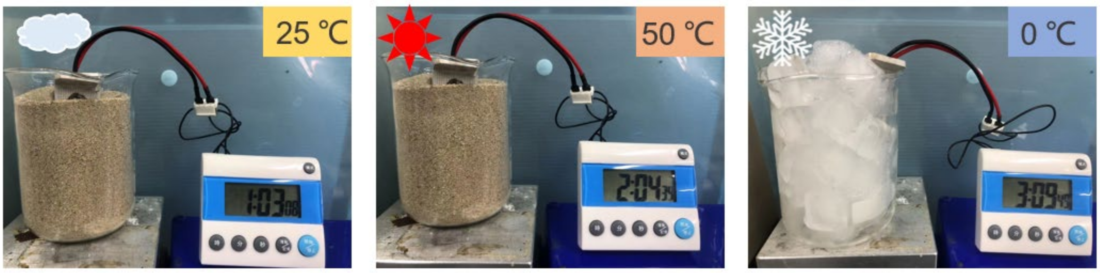

Supplementary Figure 19. Research on working under wide temperature ranges of Zn//BBQPH battery. Photographs of a timer powered by the Zn//BBQPH battery working at different temperature conditions.

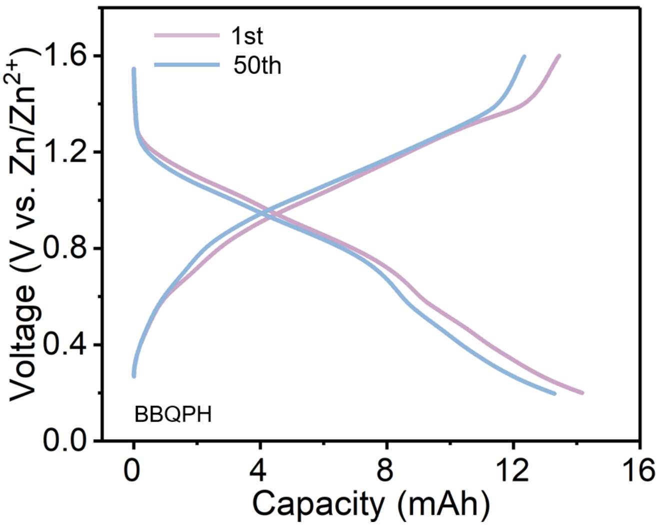

Supplementary Figure 20. The charge and discharge curves of pouch-type Zn//BBQPH battery at 0.1 A g^{-1}.

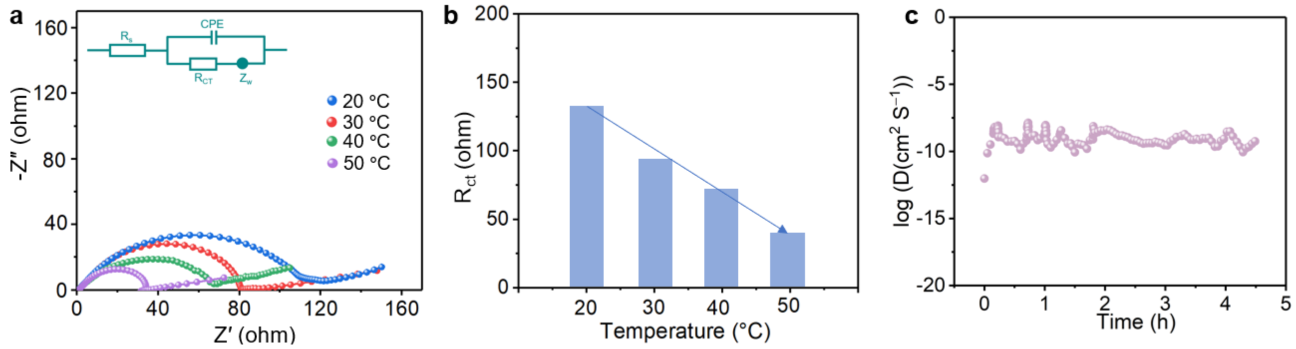

Supplementary Figure 21. The ion diffusion kinetics studies of the BBQPH cathode. (a) Electrochemical impedance spectroscopy (EIS) of BBQPH cathode at various temperatures (inset is a typical equivalent circuit, which includes the equivalent series resistance (Rs), charge transfer resistance (Rct), Warburg impedance (Zw) and constant phase angle element (CPE)) and (b) corresponding Rct values during protonation and zincification. (c) The diffusion coefficient of Zn^{2+} in BBQPH.

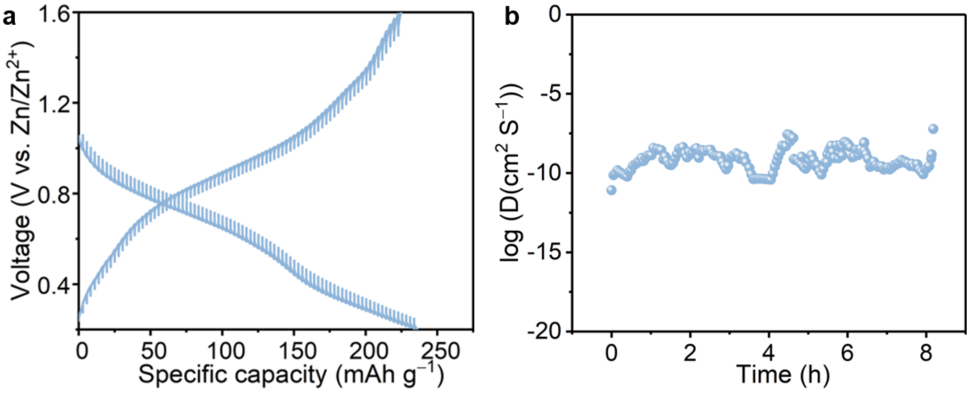

Supplementary Figure 22. The ion diffusion kinetics studies of the BBQPD cathode. (a) Galvanostatic intermittent titration technique (GITT) curve and (b) diffusion coefficient of the BBQPD cathode.

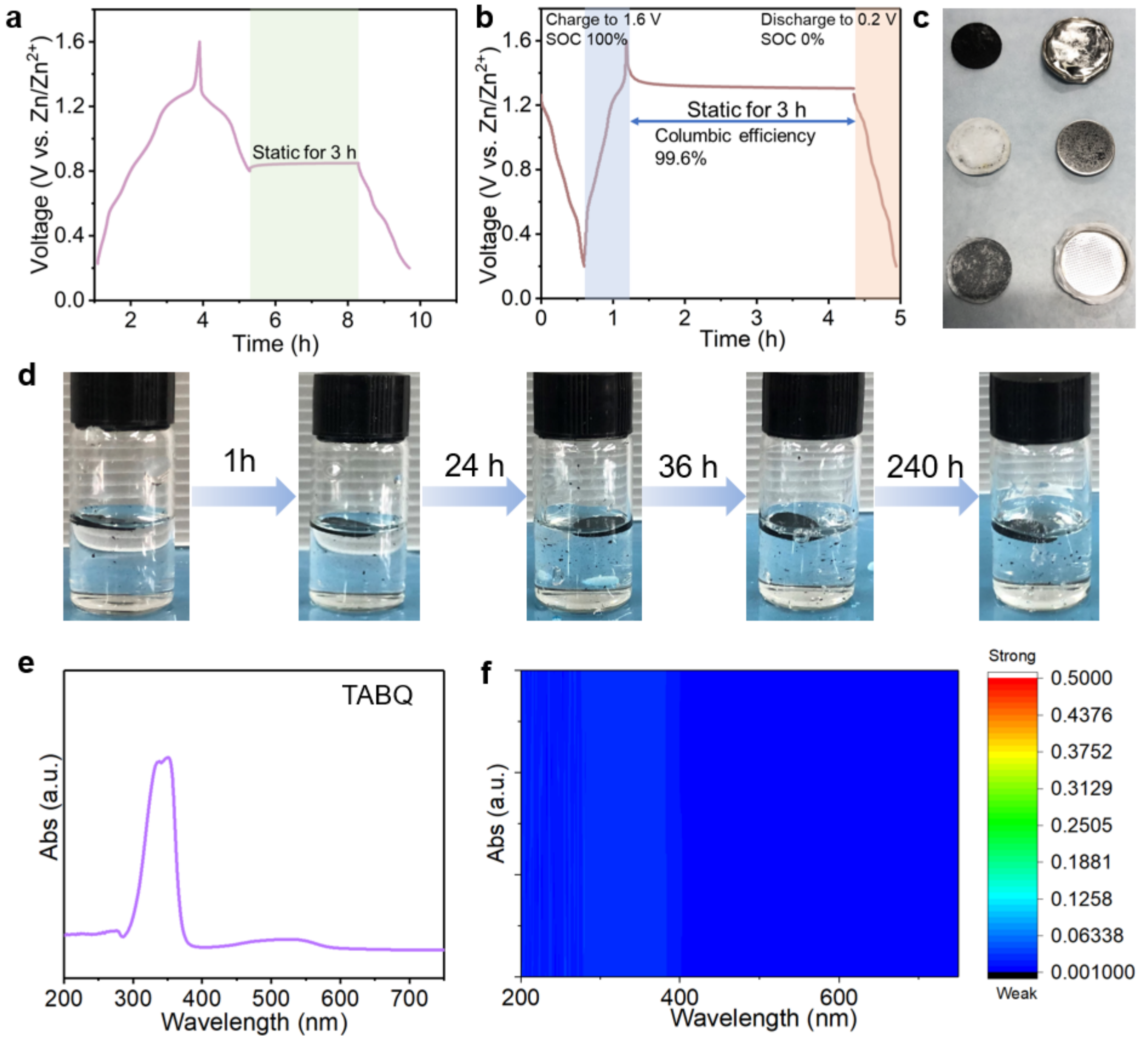

Supplementary Figure 23. Electrode stability studies. (a) The Zn/BBQPH cells in 3 M $ZnSO_4$ were first fully charged to 1.6 V at 1.0 A g^{-1} (based on active materials of cathode), and then the cells were rested at 50 % stage of charge (SOC) for 3.0 h, followed by full discharging. (b) The Zn/BBQPH battery was first fully charged to 1.6 V at 2.0 A g^{-1}, and then the cells were rested at 100 % stage of charge (SOC) for 3.0 h, followed by full discharging. (c) The photograph of the disassembled cell after 1000 cycles. (d) The photographs of the electrode immersed in the electrolyte at different times. (e) UV-visible spectroscopy of TABQ. (f) The ex situ UV-visible spectroscopy of electrolytes in Zn/BBQPH cells during the charge/discharge process.

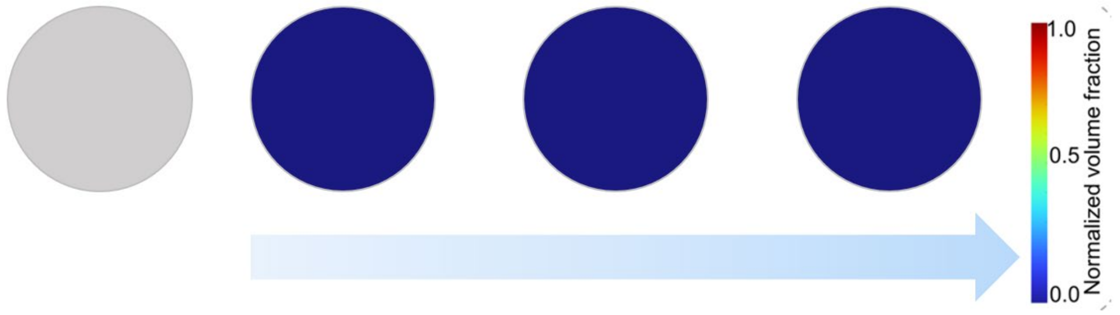

Supplementary Figure 24. **Computational fluid dynamics simulations for BBQPD.**

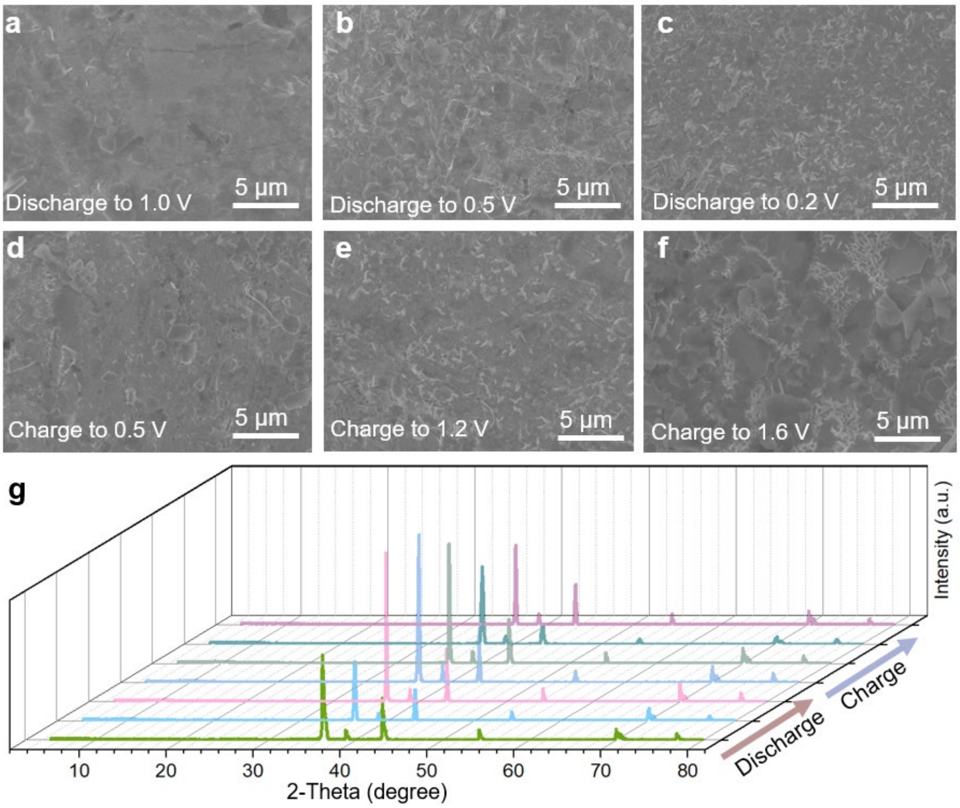

Supplementary Figure 25. The SEM and XRD characterizations of Zn anodes at six specific cut-off operating voltage. (a)-(f) The SEM images of the Zn anode for the Zn//BBQPH battery at six specific cut-off operating voltage during (dis)charge process and (g) the corresponding XRD patterns. Note: The SEM and XRD characterizations were conducted to survey the evolution of morphology and composition for Zn anode at six specific cut-off operating voltage during (dis)charging. The different discharged/charged Zn anodes exhibit rough surface configuration, which originates from the reversible plating/stripping reaction of Zn during round-trip (dis)charged process. Meanwhile, there are no impurity peaks detected in XRD patterns. These results demonstrate the good stability of Zn plating/stripping reaction in Zn//BBQPH system.

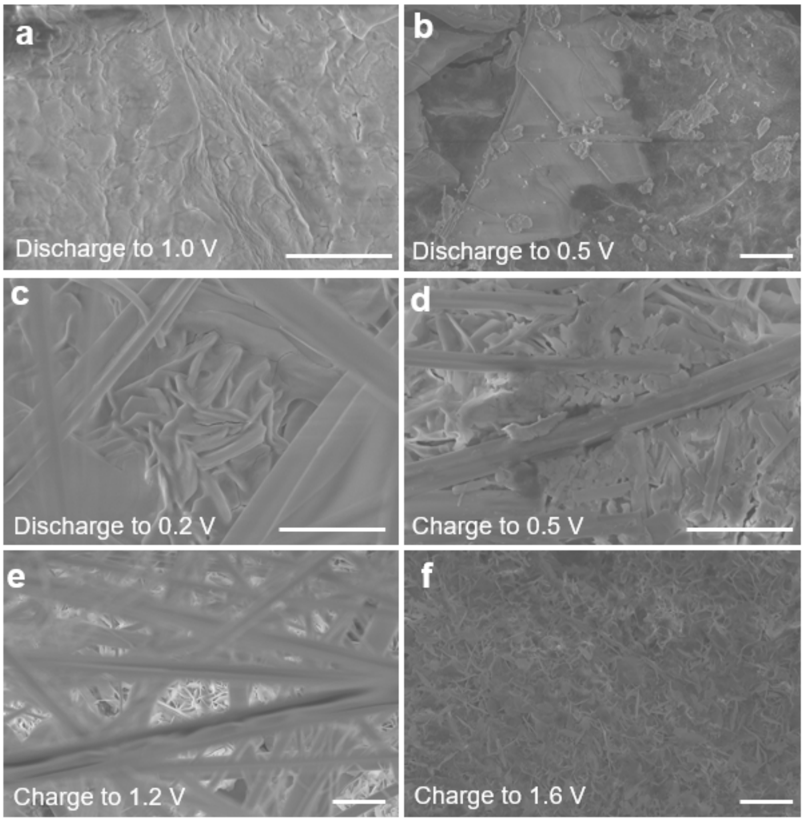

Supplementary Figure 26. The SEM characterizations of BBQPH composite cathode at six specific cut-off operating voltage. (a-f) The SEM images of the BBQPH cathode at different voltage states during the discharge/charge process. The scale bar stands up 5 μm.

Note: The SEM characterizations were conducted to survey the evolution of morphology and composition for BBQPH composite cathode at six specific cut-off operating voltage during (dis)charging. The flake-like configuration is generated on the BBQPH composite cathode as the discharge progresses, which originates from the formation of $Zn_4SO_4(OH)_6{\cdot}xH_2O$.

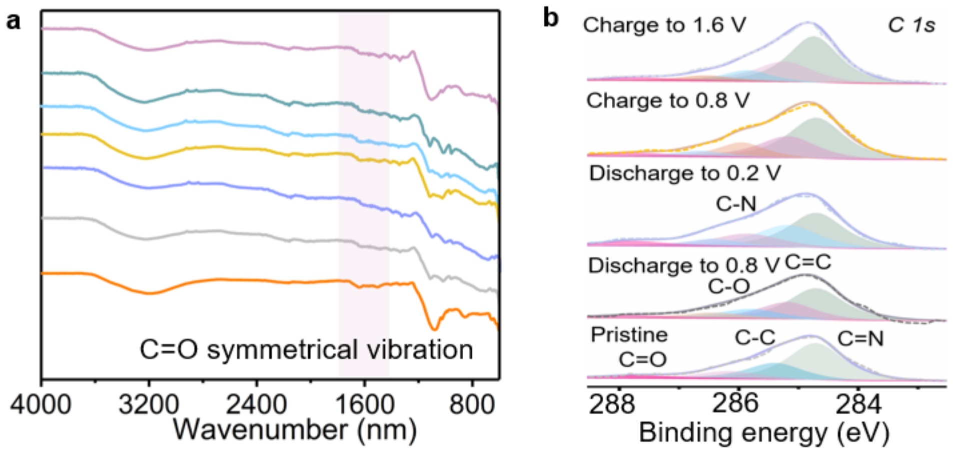

Supplementary Figure 27. Analysis of charge storage mechanisms for BBQPH. The ex situ FTIR (a) and *C 1s* XPS spectrum (b) of BBQPH composite cathodes at specific cut-off operating voltage during the discharge/charge process.

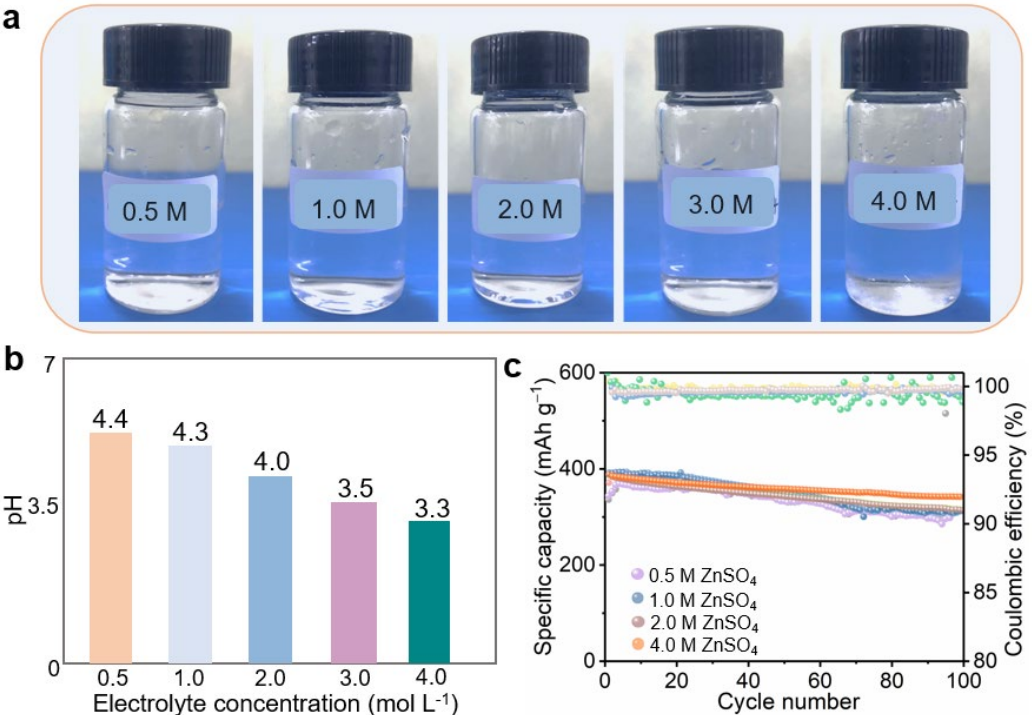

Supplementary Figure 28. **Electrochemical performance of Zn//BBQPH batteries at different electrolyte concentrations.** (a) The photographs of the 0.5, 1.0, 2.0, 3.0 and 4.0 M $ZnSO_4$ electrolytes. (b) The corresponding pH of the electrolytes. (c) The Coulombic efficiencies and cycling performances of Zn//BBQPH batteries using different concentrations of electrolytes.

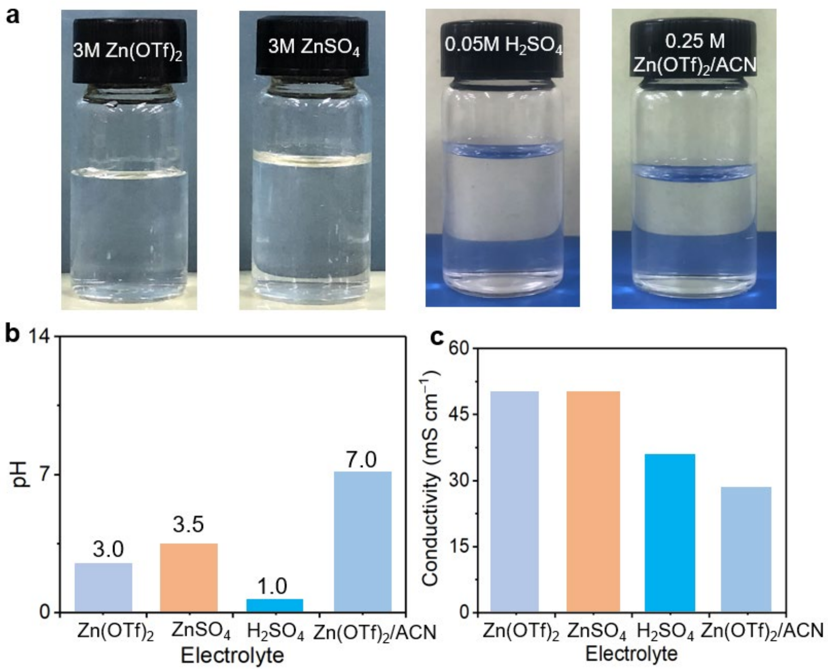

Supplementary Figure 29. Comparison of physical properties of the electrolytes. (a) The optic-photograph and corresponding (b) The pH and (c) ion-conductivities of the different electrolytes.

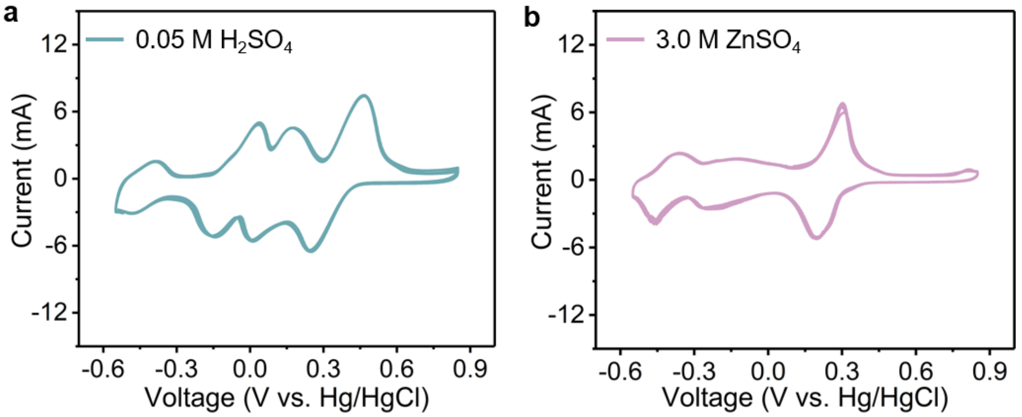

Supplementary Figure 30. Study of charge storage mechanism of BBQPH based on CV measurement. CV curves of BBQPH at 1.0 mV s^{-1} in (a) 0.05 M H_2SO_4 aqueous electrolyte and (b) 3 M $ZnSO_4$ aqueous electrolyte measured by three-electrode systems, respectively.

Note: The dotted line represents the corresponding CV curves in 0.05 M H_2SO_4 solution after being shifted to imitate its acting in a dilute H_2SO_4 electrolyte with a pH value of about 3.7.

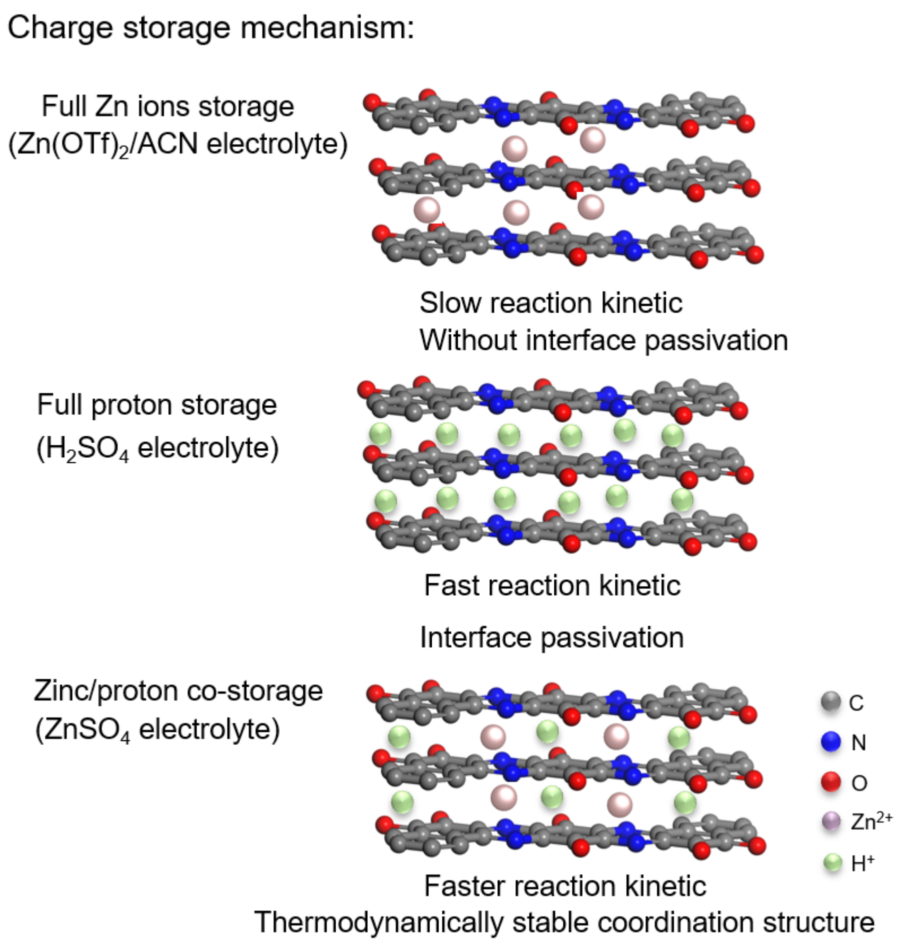

Supplementary Figure 31. The charge storage mechanism of BBQPH in the different electrolytes.

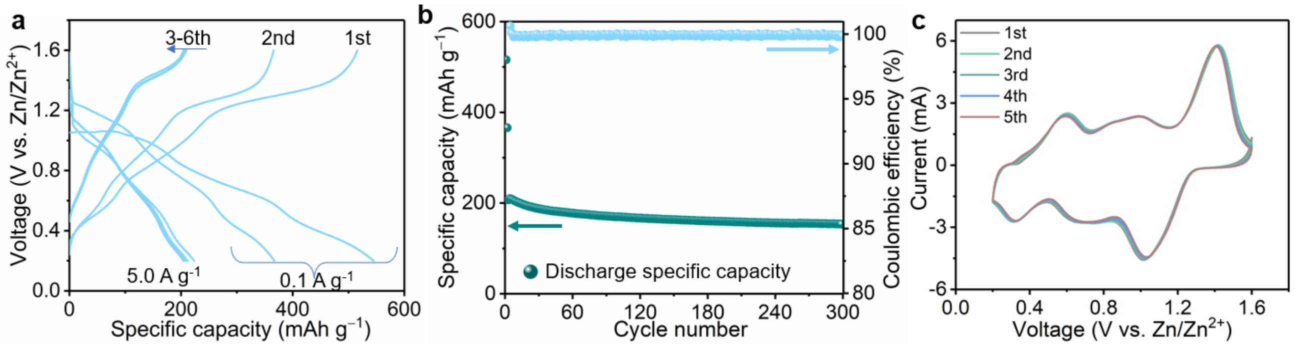

Supplementary Figure 32. Electrochemical behavior of Zn//BBQPH battery using 0.5 M Zn(OTf)$_2$/acetonitrile (ACN) electrolyte. (a) The charging and discharging curves and (b) corresponding cycling performance of Zn//BBQPH battery using 0.5 M Zn(OTf)$_2$/ACN electrolyte. (c) The CV curves of Zn/BBQPH battery employing 0.5 M Zn(OTf)$_2$/ACN electrolyte

Note: The Zn/BBQPH battery using 0.5 M Zn(OTf)$_2$/ACN electrolyte can still delivers satisfactory specific capacity at low current density (0.1 A g^{-1}). However, the battery demonstrates lower specific capacity at a high current density (5.0 A g^{-1}), which can be attributed to the slow reaction kinetics for Zn^{2+} storage. Despite this, the Zn/BBQPH battery using 0.5 M Zn(OTf)$_2$/ACN still shows the best electrochemistry performances compared with organic material for pure zinc ion storage reported.

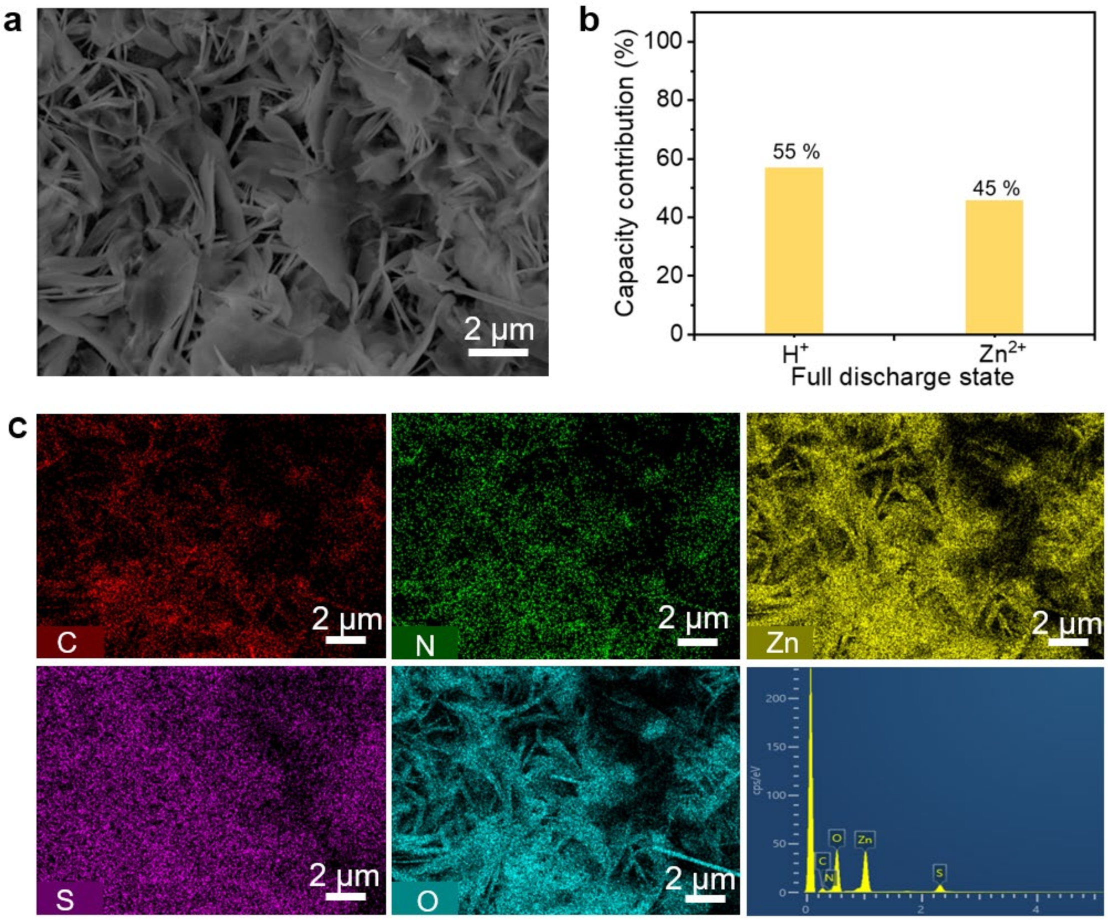

Supplementary Figure 33. Compositional and morphological structural analysis for BBQPH electrode after discharged. (a) The SEM image of BBQPH after full discharge and (b) contribution ratio of Zn^{2+} and proton of Zn//BBQPH battery using 3.0 M $ZnSO_4$ electrolyte. (c) EDS mapping of the BBQPH electrode after full discharge.

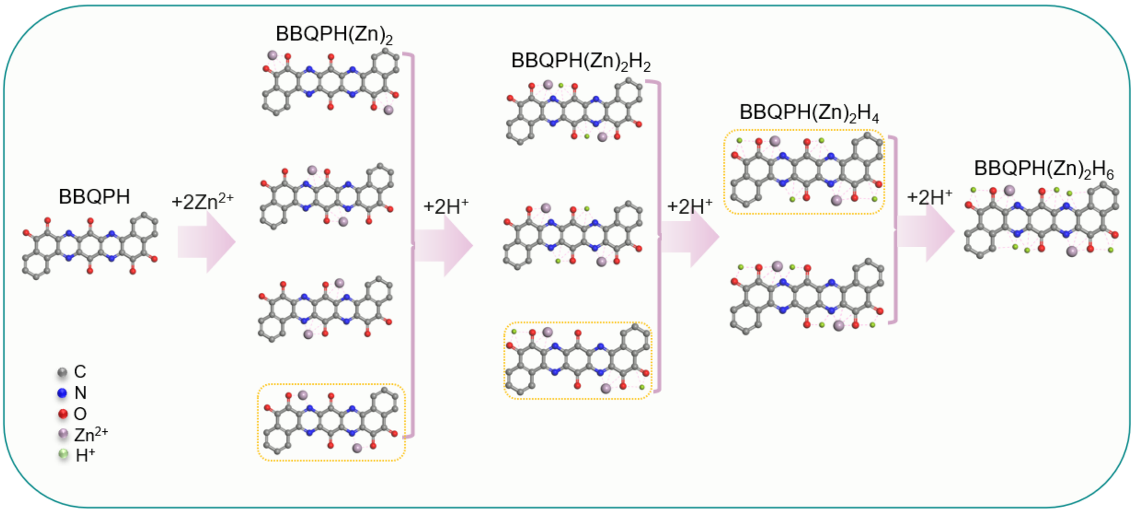

Supplementary Figure 34. The optimized Zn^{2+}/H^{+} co-storage reaction path of BBQPH.

Note: We adopted a para-equivalent ion insertion method due to the special center rotational symmetry of BBQPH to simplify DFT calculations.

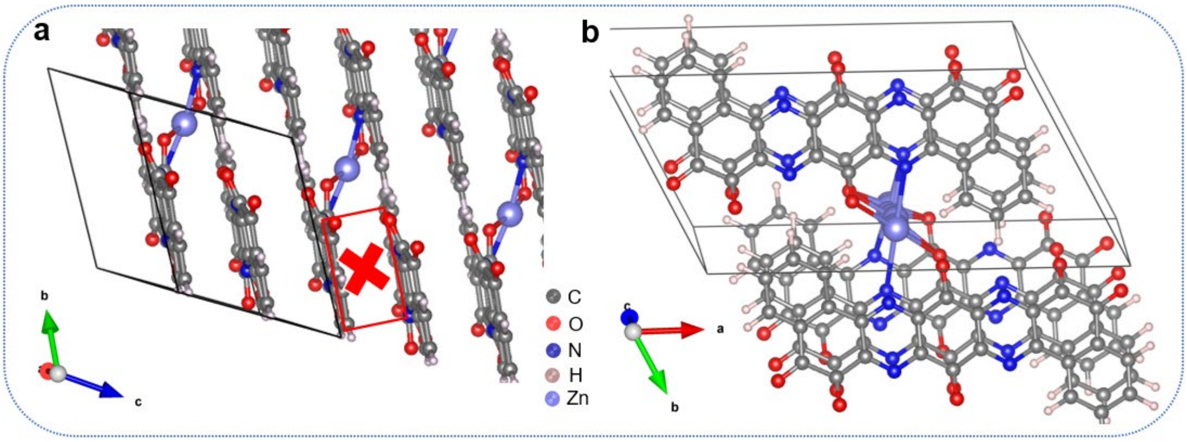

Supplementary Figure 35. The energy barrier of Zn^{2+} migration along the different directions in BBQPH. The Zn^{2+} migration along the interlayered molecular layers (P2) (a) and nanoporous channels (P1) (b).

Note: The energy barrier of Zn^{2+} migration along the molecular layers is greater than 10 eV, which can be considered to be that Zn^{2+} hardly migrates between layers due to strong intermolecular π-π forces. However, This Zn^{2+} can easily transport along the nanopores (1.42 eV) that are constructed by the molecule's orderly assembly.

Supplementary Table 1. Elemental analysis of BBQPH electrode after discharge. (a) The proportion of elemental mass after discharge in the BBQPH electrode is based on (b) XPS spectral results.

a

Element	Wt%
C	11.3
N	1.5
O	14.2
S	4.1
Zn	68.9

b

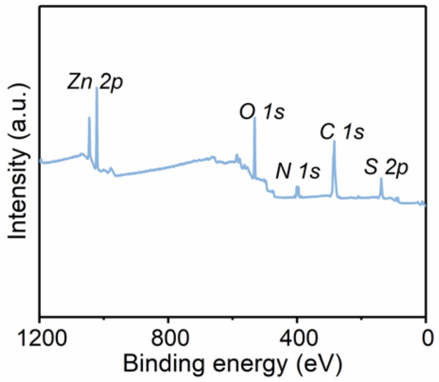

Supplementary Table 2. The atomic coordinates of the simulated BBQPH.

**Space-group P1<br>Cell a=17.204 Å b=9.163 Å c=3.553 Å V= 453.8 Å^3<br>α=114.0° γ= 94.4° β =63.4°**			
Atom name	x	y	z
H1	0.93849	0.52565	0.85398
H2	0.87265	0.83091	0.86998
H3	0.71476	0.97966	0.78395
H4	0.62317	0.82436	0.69633
H5	0.02898	0.30533	0.23044
H6	0.09504	-0.00021	0.21286
H7	0.25266	0.85166	0.30232
H8	0.34405	0.00786	0.39473
C1	0.39218	0.50176	0.48599
C2	0.42612	0.33388	0.50907
C3	0.51949	0.24075	0.58084
C4	0.57504	0.33006	0.60472
C5	0.54106	0.49804	0.58206
C6	0.44767	0.59126	0.51059
C7	0.70831	0.32778	0.69178
C8	0.67448	0.49752	0.67117
C9	0.259	0.50371	0.39657
C10	0.29279	0.33422	0.41839
C11	0.80079	0.23912	0.77016
C12	0.86146	0.31896	0.74821
C13	0.81963	0.49992	0.75547
C14	0.72913	0.58617	0.71728
C15	0.16677	0.59174	0.31481
C16	0.10593	0.51248	0.33809
C17	0.14773	0.33157	0.33157
C18	0.23815	0.24556	0.37168
C19	0.86998	0.59022	0.81258
C20	0.83251	0.76257	0.82579
C21	0.7438	0.84646	0.78167
C22	0.69231	0.75922	0.72833
C23	0.09747	0.24101	0.27279
C24	0.13502	0.06858	0.25934
C25	0.22362	-0.01505	0.30526
C26	0.27498	0.07259	0.36109

N1	0.65879	0.2471	0.66149
N2	0.5902	0.57997	0.61553
N3	0.30855	0.58437	0.42729
N4	0.37701	0.25185	0.47493
O1	0.42039	0.72851	0.45421
O2	0.54677	0.10355	0.63746
O3	0.82704	0.11505	0.87561
O4	0.94055	0.232	0.74094
O5	0.14103	0.71476	0.20579
O6	0.02686	0.59956	0.34483

Supplementary Table 3. Comparison of rate capacity, energy density, and cycling performance of recently reported organic molecule cathode materials for ZOBs in the literatures. All the electrochemical performances were tested at 25 °C.

Sample structures	Rate capacity (mAh g^{-1})	Cycling capacity retention	Energy density (Wh kg^{-1})	Electrolyte	Mass loading (mg cm^{-2})	Refs.
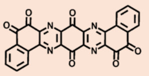	393.6@8 A g^{-1}	95%, 1000 cycles, 5 A g^{-1}	355	3 M $ZnSO_4$	1.0-2.5	This work
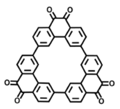	210@0.15A g^{-1}	99%, 500 cycles, 0.15 A g^{-1}	N/A	Zn-TFMS-PC	2.0	2
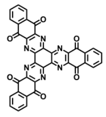	145@20 A g^{-1}	82%, 1000 cycles, 10 A g^{-1}	N/A	3 M $ZnSO_4$	1.0-1.5	3
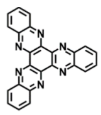	150@5 A g^{-1}	93%, 5000 cycles, 5 A g^{-1}	N/A	2 M $ZnSO_4$	1.5	4
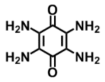	213@5 A g^{-1}	83%, 1000 cycles, 5 A g^{-1}	N/A	1 M $ZnSO_4$	1.3	1
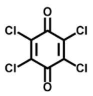	68@2.2 A g^{-1}	94%, 1000 cycles, 1.1 A g^{-1}	286	4M $Zn(BF_4)_2$	1.0-2.0	5
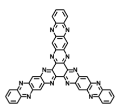	131@60 A g^{-1}	93%, 30000 cycles, 30 A g^{-1}	153.9	2 M $ZnSO_4$	1.0-2.0	6
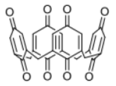	172@1 A g^{-1}	87%, 1000 cycles, 0.5 A g^{-1}	220	3 M $Zn(CF_3SO_3)_2$	2.5 -10	7
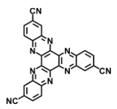	190@20 A g^{-1}	91%, 5800 cycles, 5 A g^{-1}	149	2 M $ZnSO_4$	1.0	8
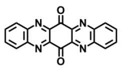	182@2 A g^{-1}	92%, 250 cycles, 0.05 A g^{-1}	282	1 M $ZnSO_4$	3.0-4.0	9

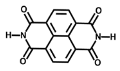	120@1C	93.5%, 1000 cycles, 1C	N/A	1 M $ZnSO_4$	1.7-2.8	10
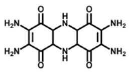	148@10 A g^{-1}	82%, 3000 cycles, 10 A g^{-1}	N/A	1 M $ZnSO_4$	1.2	11
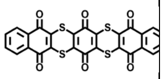	208@30 A g^{-1}	99%, 2000 cycles, 10 A g^{-1}	N/A	3.5 M $Zn(ClO_4)_2$	1.0-2.0	12
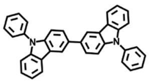	80@0.4 A g^{-1}	96%, 1000 cycles, 0.5 A g^{-1}	N/A	1m $Zn(N(SO_2CF_3)_2)_2$	3.0-5.0	13
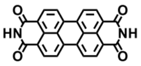	105@8 A g^{-1}	81%, 500 cycles, 8 A g^{-1}	60	3 M $ZnSO_4$	2.0-3.0	14
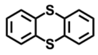	66@20 A g^{-1}	82%, 8000 cycles, 1 A g^{-1}	67	1 M $Zn(CF_3SO_3)_2$	1.0-2.0	15
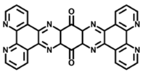	161@20 A g^{-1}	92%, 7500 cycles, 10 A g^{-1}	348	1 M $ZnSO_4$ + 2 M Na_2SO_4	1.0-2.0	16
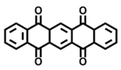	90@2 A g^{-1}	85%, 3000 cycles, 1 A g^{-1}	N/A	1 M $Zn(CF_3SO_3)_2$	1.0-2.0	17
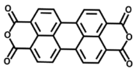	105@8 A g^{-1}	81%, 500 cycles, 8 A g^{-1}	60	2 M $ZnCl_2$	1.3	18
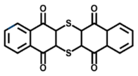	100@2 A g^{-1}	84%, 23000 cycles, 2 A g^{-1}	126	2 M $ZnSO_4$	5.0	19
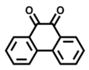	111@10 A g^{-1}	96%, 36000 cycles, 5 A g^{-1}	86.5	2 M $ZnSO_4$	2.0-3.0	20

Supplementary References:

1. Lin Z, Shi HY, Lin L, Yang X, Wu W, Sun X. A high capacity small molecule quinone cathode for rechargeable aqueous zinc-organic batteries. *Nat. Commun.* **12**, 4424 (2021).
2. Wang N*, et al.* Zinc-Organic Battery with a Wide Operation-Temperature Window from -70 to 150 degrees C. *Angew. Chem. Int. Ed.* **59**, 14577-14583 (2020).
3. Chen Y*, et al.* Two-Dimensional Organic Supramolecule via Hydrogen Bonding and pi-pi Stacking for Ultrahigh Capacity and Long-Life Aqueous Zinc-Organic Batteries. *Angew. Chem. Int. Ed.* **61**, e202116289 (2022).
4. Tie Z, Liu L, Deng S, Zhao D, Niu Z. Proton Insertion Chemistry of a Zinc-Organic Battery. *Angew. Chem. Int. Ed.* **59**, 4920-4924 (2020).
5. Sun T*, et al.* An ultralow-temperature aqueous zinc-ion battery. *J. Mater. Chem. A* **9**, 7042-7047 (2021).
6. Li S*, et al.* Design and Synthesis of a π-Conjugated N-Heteroaromatic Material for Aqueous Zinc-Organic Batteries with Ultrahigh Rate and Extremely Long Life. *Adv. Mater.* e2207115 (2022).
7. Zhao Q*, et al.* High-capacity aqueous zinc batteries using sustainable quinone electrodes. *Sci. Adv.* eaao1761 (2018).
8. Ye Z*, et al.* High-rate aqueous zinc-organic battery achieved by lowering HOMO/LUMO of organic cathode. *Energy Storage Mater.* **37**, 378-386 (2021).
9. Gao Y, *et al.* A high-performance aqueous rechargeable zinc battery based on organic cathode integrating quinone and pyrazine. *Energy Storage Mater.* **40**, 31-40 (2021).
10. Na M, Oh Y, Byon HR. Effects of Zn^{2+} and H^{+} Association with Naphthalene Diimide Electrodes for Aqueous Zn-Ion Batteries. *Chem. Mater.* **32**, 6990-6997 (2020).
11. Lin L*, et al.* A semi-conductive organic cathode material enabled by extended conjugation for rechargeable aqueous zinc batteries. *Energy & Environ. Sci.* **16**, 89-96 (2023).
12. Sun T, Zhang W, Nian Q, Tao Z. Molecular Engineering Design for High-Performance Aqueous Zinc-Organic Battery. *Nano-Micro. Lett.* **15**, 36 (2023).
13. Mittal U, Colasuonno F, Rawal A, Lessio M, Kundu D. A highly stable 1.3 V organic cathode for aqueous zinc batteries designed in-situ by solid-state electrooxidation. *Energy Storage Mater.* **46**, 129-137 (2022).

14. Liu N, *et al.* Building High Rate Capability and Ultrastable Dendrite-Free Organic Anode for Rechargeable Aqueous Zinc Batteries. *Adv. Sci.* **7**, 2000146 (2020).

15. Cui H, *et al.* High-Voltage Organic Cathodes for Zinc-Ion Batteries through Electron Cloud and Solvation Structure Regulation. *Angew. Chem. Int. Ed.* **61**, e202203453 (2022).

16. Peng H, *et al.* N-Heterocycles Extended π-Conjugation Enables Ultrahigh Capacity, Long-Lived, and Fast-Charging Organic Cathodes for Aqueous Zinc Batteries. *CCS Chemistry*, 1-13 (2022).

17. Wu M, *et al.* Long-Life Aqueous Zinc–Organic Batteries with a Trimethyl Phosphate Electrolyte and Organic Cathode. *ACS Sustainable Chem. Eng.* **11**, 957-964 (2023).

18. Zhang H, Fang Y, Yang F, Liu X, Lu X. Aromatic organic molecular crystal with enhanced π–π stacking interaction for ultrafast Zn-ion storage. *Energy & Environ. Sci.* **13**, 2515-2523 (2020).

19. Wang Y, *et al.* Binding Zinc Ions by Carboxyl Groups from Adjacent Molecules toward Long-Life Aqueous Zinc-Organic Batteries. *Adv. Mater.* **32**, e2000338 (2020).

20. Yang B, Ma Y, Bin D, Lu H, Xia Y. Ultralong-Life Cathode for Aqueous Zinc-Organic Batteries via Pouring 9,10-Phenanthraquinone into Active Carbon. *ACS Appl. Mater. Interfaces* **13**, 58818-58826 (2021).
